# Picture Perfect Pups: How Do Attributes of Photographs of Dogs in Online Rescue Profiles Affect Adoption Speed?

**DOI:** 10.3390/ani10010152

**Published:** 2020-01-16

**Authors:** Mizuho Nakamura, Navneet Dhand, Bethany J. Wilson, Melissa J. Starling, Paul D. McGreevy

**Affiliations:** Sydney School of Veterinary Science, Faculty of Science, The University of Sydney, Sydney, NSW 2006, Australia; mnak1804@uni.sydney.edu.au (M.N.); navneet.dhand@sydney.edu.au (N.D.); bethany.wilson@sydney.edu.au (B.J.W.); mjstarling@fastmail.com.au (M.J.S.)

**Keywords:** dogs, welfare, adoption, rescue, morphology, breed, photographs

## Abstract

**Simple Summary:**

This study examined the photographs of 8332 dogs on the website of PetRescue, Australia’s largest online directory of animals in need of adoption. By investigating a range of photographic attributes, we revealed significant associations between several variables and length of stay (LOS) online, indicating visual characteristics that prospective owners find appealing when looking at rescue dogs online. The photographic attributes associated with the shortest LOS in the current study were mouths closed, a black coat colour, floppy ears and being photographed in a kennel structure. Several of these results contrast with those from previous studies, such as that dogs generally have a better chance of being adopted if they look as though they are owned, rather than living in a rescue shelter. Our results imply that many users of pet adoption sites may actively favour dogs who appear to need their help. We suggest that our results may reveal more about the users of these sites than general human responses to photographs of dogs per se.

**Abstract:**

To increase the public’s awareness of and exposure to animals needing homes, PetRescue, Australia’s largest online directory of animals in need of adoption, lists all currently available animals from rescue and welfare shelters nationwide. The current study examined the photographs in the PetRescue online profiles of the three most common breeds within these data, namely, Staffordshire bull terriers (n = 3988), Labrador retrievers (n = 2246), and Jack Russell terriers (n = 2088), to identify the inferred preferences of potential adopters. By investigating the attributes of these photographs, we were able to identify visual risk factors associated with protracted lengths of stay (LOS). The longest stays were associated with dogs with erect ears and those photographed in a natural environment, i.e., 18.32 days and 19.57 days, respectively. Dogs photographed in a kennel and with mouths closed had the shortest LOS, i.e., 11.54 d and 14.44 d, respectively. Heightened awareness of the roles of photographic attributes in generating interest among potential adopters may increase the speed of adoption by guiding the creation of online profiles and selection of photos to optimise the promotion of dogs at risk of long stays.

## 1. Introduction

Dogs have been living alongside humans for thousands of years, and have been subject to artificial selection to assist with various roles including hunting, guarding and the herding of livestock. They continue to work in these roles, but now also occupy niches as companions to humans. They have also become service dogs for the visually impaired and physically disabled, therapy dogs to assist those with mental health disorders [1] and detectors of drugs and explosive materials for various law enforcement and military agencies. Since domestication, dogs have become integrated into our lifestyles chiefly because of their sociability, behavioural flexibility and ability to engage with communicative cues from humans [2,3]. Indeed, it has been suggested that this ability to read human signals has advanced with domestication. Dogs can communicate with humans better than wolves, and, in parallel, have developed outstanding social skills to work for and cooperate with humans [4]. These attributes enhance their suitability for various working and companionship roles.

Despite the benefits that dogs can bring to human carers and coworkers, thousands of companion dogs, in Australia alone, are abandoned and relinquished to welfare shelters every year [5]. Welfare shelters may perform behaviour assessments to identify dogs that can readily and safely become members of the community once more, thus increasing the likelihood of successful adoption [5,6]. However, there may be other means to increase the likelihood of adoption for many of these dogs. PetRescue, Australia’s largest online directory of animals needing a home, invites shelters and rescue groups throughout Australia to list on its website the animals that they have available. This provides a centralised collection of thousands of animals in need of adoption so that potential adopters can save time and effort by searching a single online portal instead of visiting many websites. The length of time animals are listed on PetRescue before being adopted can be described as their length of stay (LOS). Dogs with an extended LOS or suboptimal adoption success may be considered to be of low interest or appeal to those visiting the website, and this, in turn, may indicate the dogs’ level of appeal to the general public. 

Fratkin and Baker [7] reported the possibility that some potential adopters may select their pet solely on its visual characteristics. Photographs are used in online profiles, acknowledging that physical features play a significant role in the appeal of companion animals to members of the public [7]. These authors [7] investigated the effects of coat colour (black or yellow) and ear shape (floppy-eared or pointy-eared) in dog photographs (n = 124) on human perceptions of dog personality. They also used a personality dimension model validated for humans to examine the dogs’ perceived agreeableness, conscientiousness, emotional stability, extraversion and openness to experience. The study found that participants rated yellow dogs significantly higher than black dogs in the dimensions of agreeableness, conscientiousness and emotional stability; and rated floppy-eared dogs higher than pointy-eared in agreeableness and emotional stability [7]. Similarly, a study by Blecker et al. [8] found that pedestrians perceived pale-coloured dogs as being friendlier than dark-coloured dogs. Siettou et al. [9] investigated the influence of both innate factors (sex and coat colour) and acquired attributes (behavioural issues and training levels) on consumer choices when adopting dogs. A mixture of the following factors were found to have a positive influence on adoption: short coat length, purebred status and dogs being friendly towards children, other dogs and other animals. Protopopova and Wynne [10] found that visitor behaviour at a shelter was associated with dog-related variables such as being young (less than 4 months) and relatively small in size, as well as having a light coat colour and long coat length. Specifically, they found that the number of visits to dogs in kennels with the above attributes were relatively higher, resulting in potential adopters taking the dogs out of the kennels to spend more time with them. In contrast, it is recognised that several factors can have a negative influence on adoption, including the reported need for further training and signs of behavioural issues [9]. These outcomes have been reported repeatedly in the literature [9,10,11,12,13,14,15,16].

As certain phenotypic characteristics, such as black coat colour and erect ears, were thought to be perceived negatively, Fratkin and Baker [7] proposed the potential benefits of different methods of advertising dogs with these characteristics, so that low-appeal animals are not overlooked by prospective owners. A study by Thorn and Mitchell [15] investigated the preferences of potential adopters by focussing on five factors which are evident in online photographs (n = 100) (wearing a collar, being in a kennel, sitting on a chair, presence of a toy and identified as a surrendered animal) and explored how these influence the selection of shelter dogs. It showed that dogs wearing a collar and photographed outside of kennels appeared to have been favoured by prospective owners [15]. 

Phenotypic characteristics have also been associated with the likelihood of adoption in previous studies [11,12]. Furthermore, Waller et al. [17] showed that having large eyes relative to the rest of the face was associated with cuteness. Their study investigated whether facial muscle contraction was a determinant of canine adoption rates from a shelter. Photographs of dogs (n = 86) were digitally manipulated to exaggerate specific facial features. This process revealed that dogs with evidence of inner brow movement had greater appeal than dogs that did not [17].

The current study investigated the visual attributes of animals on the PetRescue website to determine which were associated with LOS. The goal of the study was to reveal features of photographs in online dog profiles that may appeal to adopters by identifying relationships between speed of adoption and various attributes of dogs’ photographs. A potential benefit of this information is that animals of potentially low appeal can be presented online in ways that maximise their appeal, and thus boost their chances of adoption.

## 2. Materials and Methods 

### 2.1. Pilot Study for Web Resource Development

A pilot study of a web-based scoring system for photographic attributes was conducted to ensure reliable and consistent results. Twenty-five photographs of each of the four breeds of initial interest (100 dogs in total) were chosen at random. Participants volunteered to take part in the scoring of photographs. Participants in this pilot study included PetRescue staff members and volunteers who consented to take part after PetRescue advertised this research and pilot study on social media. This process tested for concordance of the scoring system, and the feedback received during the pilot study ensured that the final suite of online questions about photographs was easy to understand for people with little prior knowledge of canine characteristics.

**Figure 1:**
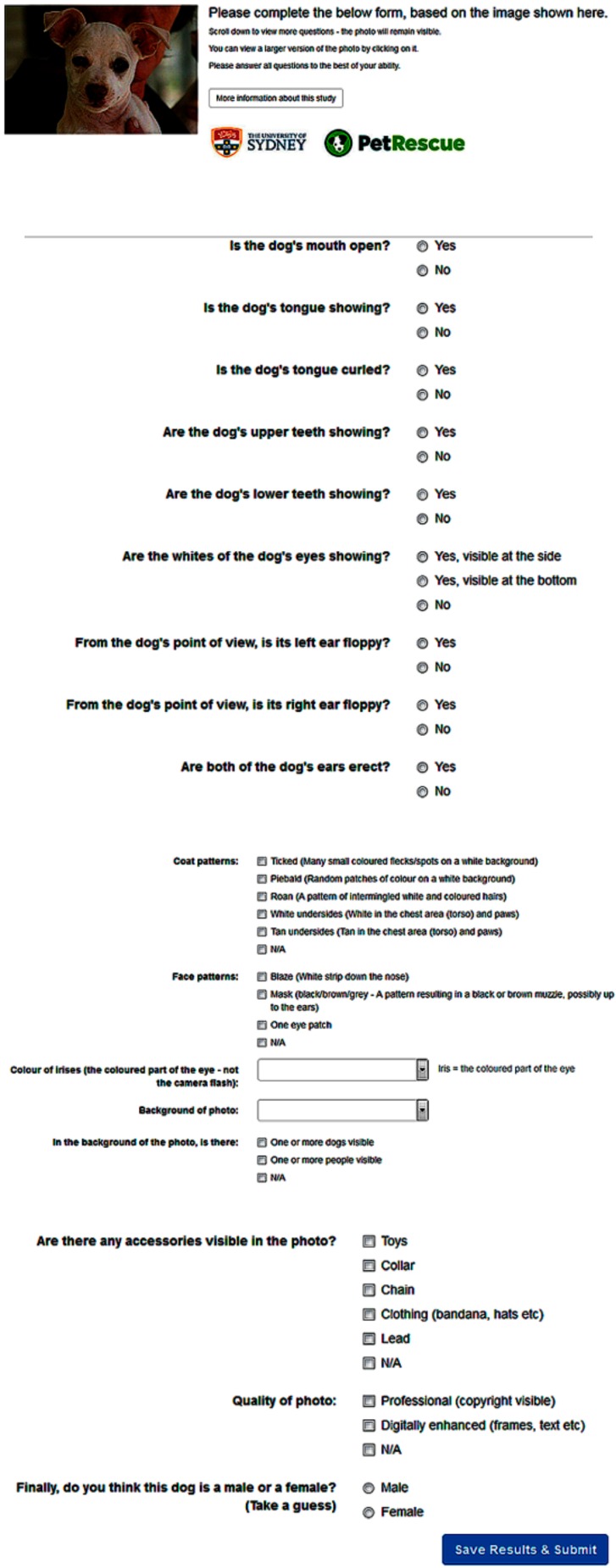
Screen shot of web form posted online on the PetRescue social media for online selection of data about photographs of dogs (http://thinklateral.com.au/dog/index.cfm).

The scoring system underwent numerous refinements and was finally developed as a web-based form (Figure 1). This system facilitated the collection of data from volunteers who were asked to read the information regarding the research before commencing. Human Research Ethics Committee approval was not requested for this volunteer activity, mediated by PetRescue. The form explored relevant photographic attributes by primarily using questions requiring a binary response. The questions about some attributes (such as coat colour, colour of irises and background of the photograph) were structured so that only mutually-exclusive answers could be submitted. Meanwhile, questions about other attributes (such as facial pattern, coat patterns and accessories in the photograph) were structured so that multiple answers could be selected.

### 2.2. Main Study

#### 2.2.1. Selection

The sample population for this study was all dogs listed on the PetRescue website from 2004 (when PetRescue was founded) until late-March, 2013 (the commencement of the current study). Over this nine-year period, 122,634 dogs were listed on the website. The date listed (i.e., when the dog was added to the website) and date removed (i.e., when the dog was adopted) by the individual welfare groups were used to calculate LOS and, by inference, adoption speed. As this study investigated variables relative to LOS and focused solely on rehomed dogs, we excluded “removed” dogs that had been removed from the website by the contributing rescue groups as distinct from “rehomed” and “active” dogs that were newly listed and had not yet been adopted from the dataset, leaving a pool of 101,397. Also, numerous dogs had data missing on LOS, and these were also excluded. After these dogs had been excluded, the total number of dogs available for this study was 70,733.

Photographs of the four most common breeds (n = 9964) within these data—i.e., Staffordshire bull terrier (n = 4128), Australian cattle dog (n = 1364), Labrador retriever (n = 2326) and Jack Russell terrier (n = 2146)—were selected for further analysis. Dogs posted as crosses of these four breeds (e.g., Kelpie x Australian cattle dog) were included. All photos were screened against a set of selection criteria that were created for this study, i.e., no blurry photographs, ensuring dogs had both eyes open, a minimum photo size of 250 by 250 pixels, and having both ears visible. A large number of Australian cattle dogs were excluded from the study as a result of this process, such that only 14 remained in the final cohort for the study. Because this represented only approximately 1% of the Australian cattle dogs among the 70,733 animals available, it was considered highly unlikely that these photos would be sufficiently representative, and so the scores for this breed were not included in further analysis, leaving 8322 dogs: Jack Russell terriers (n = 2088; 25.1%), Labrador retrievers (n = 2246; 27.0%) and Staffordshire bull terriers (n = 3988; 47.9%).

#### 2.2.2. Photographic Attribute Scoring

For each photograph, a total of 16 attributes could be scored. The photographic attributes were divided into mutable and immutable characteristics. The mutable characteristics included whether the dog’s mouth was open (tongue showing, tongue curled, upper and/or lower teeth showing), background of the photograph (nature, kennel, indoors, outdoors, other dogs or humans visible), whether the sclera was visible (sclera to the side or at the bottom), accessories in the photograph (toys, collar, lead, chain or clothing) and the quality of the photograph (professional photograph with copyright assigned, digital enhancements such as frames and texts added). The immutable characteristics included ear placement (both ears erect, left or right ear floppy from the dog’s point of view), coat colour (black, brown, yellow/golden, white, bicolour, tricolour or brindle), coat patterns (ticked, piebald, roan, tan undersides or white undersides), facial markings (blaze, mask or one eye patch), and colour of irises (brown, light brown, blue/grey, amber or wall eyes). All multicategory variables on the online form also had “Not Applicable (N/A)” as a response option. The final question of the online form asked participants to guess the sex of the animal, simply by looking at the photograph. This was an attempt to establish whether people can recognise dimorphic characteristics that have been reported in some breeds of dog [18]. A glossary of terms was also provided to participants in the study to ensure definitions and understandings were consistent. Breed-specific terminology (such as chocolate, fawn and red) for coat colour was also provided. Photographic examples were provided to illustrate the various facial patterns, coat colour and patterns of the included breeds. Neither the names nor the locations of shelters were visible to the participants.

### 2.3. Data Analysis

Descriptive analyses were conducted initially and included the calculation of summary statistics and graphical summaries of LOS and frequency tables for all photographic attributes. Data were analysed through general linear models using the linear model (lm) function of the stats package of the R statistical software version 3.6.1 [19]. The LOS, transformed toward normality using a log transformation, was set as the dependent variable for each of multiple linear models, which each included sex and breed as dependent variables to account for varying popularity among breeds and between sexes for adoption. Interaction terms for these variables with each photographic variable were initially modelled to investigate differential effects of photographic variables across breeds and sexes. The effect on length of stay was derived from the coefficient associated with the photographic dependent variable of each model. Due to the limited number of breeds represented, interactions between photographic attributes were considered beyond the scope of the study. Future work could explore whether attributes such as coat colour differentially modify the effects on LOS of other attributes, particularly mutable ones. 

## 3. Results

The median LOS was 17.89 d, with 25% of dogs being rehomed before 7.04 d and 75% being rehomed before 39.24 d. Of the three focal breeds, Labrador retrievers and crosses had the shortest median time to rehoming, i.e., 16.6 d (IQR = 28.6 d), Jack Russell terriers and crosses were intermediate with a median of 17.9 d (IQR = 33.9 d) and Staffordshire bull terriers spent the longest in shelters, with a median LOS of 18.8 d (IQR = 33.2 d).

The median LOS for female dogs (17.1 d; IQR = 30.3 d) was significantly shorter (t = −3.0174, *p* = 0.002, effect size = 0.07) than that for male dogs (18.6 d, IQR = 33.50 d). Out of 8332 scored photos, 4742 of the dog’s gender were guessed correctly, demonstrating an accuracy of only 57%.

### 3.1. Immutable Characteristics

#### 3.1.1. Ears

Volunteers coded 1772 dogs as having no floppy ears (21.3%), 488 as having one floppy ear (5.9%), and 6062 (72.8%) as having two floppy ears. In a model corrected for breed and sex, having no floppy ears was associated (t = 3.109, *p* = 0.002, effect size = 0.07) with an estimated additional 1.8 days before rehoming compared to dogs with two floppy ears, while having one floppy ear was associated (t = 3.655, *p* < 0.001, effect size = 0.08) with an estimated additional 3.7 days before rehoming. Interaction terms between breed and floppy ears, and sex and floppy ears, were nonsignificant, suggesting no indication of this symmetrical floppy-eared preference varying among the three breeds or between the sexes in the dataset. 

#### 3.1.2. Coat Colour

Bicolour (n = 818; 39.2%) was the most common colour selected for Jack Russell terriers, black was the most common colour for Labrador retrievers (n = 960, 42.7%), and brindle was the most common colour for Staffordshire bull terriers. Due to the presence of significant interaction terms between breed and coat colour, and the difficulty of selecting a single reference class appropriate to all three breeds, the effect of coat colour on each breed was modelled in a separate univariate model.

When corrected for sex, and compared to bicolours, Jack Russell terrier colours associated with significantly shorter stays were brown (t = −2.640, *p* = 0.008, effect size = 0.12), tricolour (t = −2.073, *p* = 0.038, effect size = 0.09) and golden/yellow (t = −2.564, *p* = 0.010, effect size = 0.11). The predicted average LOS for these colours were shorter than for bicolours by 4.7 d, 3.0 d and 6.8 d, respectively.

Among Labrador retrievers, no coat colours were associated with significantly longer or shorter LOS than black, the most common colour for the breed. Among Staffordshire bull terriers, black coat colours were associated with significantly shorter stays than brindle; the most common colour for the breed (t = −3.906, *p* < 0.01, effect size = 0.12). The predicted average stays LOS for black Staffordshire bull terriers was 4.0 d shorter than the median LOS for brindle dogs of the breed.

#### 3.1.3. Face Markings

Modelling of blazes, masks and an absence of face pattern revealed no significant increase or decrease in LOS when corrected for breed, sex or potential interactions.

Conversely, modelling for the presence of a single eye patch found significant effects among Labrador retrievers (and their crosses). When this breed was modelled separately, it was discovered that in Labrador retrievers, one eye patch was an advantage for female dogs, reducing their estimated average adoption time by about 6.7 d. However, for male Labrador retrievers, a single eye patch was associated with a distinct disadvantage, increasing LOS by an estimated 24.8 d (t of interaction = 2.731, *p* = 0.006, effect size = 0.12). It is worth noting that only a small number of Labrador retrievers had this eye patch marking (9 of 937 females and 8 of 1309 males), and thus care should be taken to avoid overinterpreting this finding, despite its statistical significance.

#### 3.1.4. Coat Patterns

Corrected for sex and breed, a piebald coat pattern was associated with a significantly (t = 2.725, *p* = 0.006, effect size = 0.06) longer LOS, i.e., an estimated 2.0 d longer. Corrected for sex and breed, a ticked coat pattern was associated with a significantly (t = 3.279, *p* = 0.001, effect size = 0.07) longer LOS, i.e., an estimated 4.0 d longer. Neither pattern showed significant interactions with breed or sex on LOS. White undersides significantly reduced LOS (t = −3.589, *p* < 0.001, effect size = 0.16) for Jack Russell terriers only by 4.2 d. The other coat markings (e.g., roan and tan undersides) were not associated with LOS. 

#### 3.1.5. Iris Colour

Modelling of iris colour revealed that the effect of eye colour on LOS varied by breed, although not all coefficients individually reached significance. Compared to brown, the most common iris colour in all breeds, i.e., blue/grey, were associated with shorter LOS (t = −2.085, *p* = 0.037 effect size = 0.05) across all breeds, although somewhat less so in Labrador retrievers (an estimated average 1.7 d less) compared with Jack Russell terriers (an estimated average 7.2 d less) and Staffordshire bull terriers (an estimated average of 6.0 d less). Light brown eyes were not associated with LOS in Jack Russell terriers and Staffordshire bull terriers, but in Labrador retrievers, having light brown eyes instead of brown eyes was associated with an average 2.9 d longer stay (t = 2.055 *p* = 0.04, effect size = 0.05).

### 3.2. Mutable Characteristics

#### 3.2.1. Mouth Variables

Corrected for breed and sex, having the mouth open in the photograph was significantly associated with a longer LOS (Type 3 Anova F value = 17,2765, df-1, *p* < 0.001). In Staffordshire bull terriers, this manifested as a predicted additional mean day of 7.8 d, significantly longer (t = 2.219, *p* = 0.03, effect size = 0.05) than the additional predicted 4.5 d for open-mouthed Jack Russell terriers. In Labrador retrievers, an open mouth was associated with an additional predicted 5.7 d, which was not significantly longer than in Jack Russell terriers (t = 1.845, *p* = 0.07). Corrected for sex and breed, visibility of the lower teeth added a predicted average of 5.3 d to the LOS. No significant interactions between the visibility of lower teeth and sex or breed were observed for LOS.

While overall, the tongue being visible corresponded to an increased LOS (t = 12.170, *p* < 0.01, effect size = 0.27), modelling with the mouth open variable suggest that this may be largely attributable to the presence of the open mouth because, when additively corrected for this variable as well as sex and breed, this variable was no longer significantly associated with LOS (t = 0.341, *p* = 0.73). Similarly, a curled tongue was received differently associated with an open mouth than with a closed one (t = 2.351, *p* = 0.02, effect size = 0.05). Compared to a photograph with a closed mouth and no curled tongue, estimated LOS was between 4.6 and 5.9 d shorter (t = −2.57, *p* = 0.01, effect size = 0.06) for photographs with a closed mouth and curled tongue (depending on breed). However, the effect of a curled tongue with an open mouth was much smaller, shortening stays by less than a half a day. 

#### 3.2.2. Other Face Characteristics

Corrected for breed and sex, visible sclera either at the bottom of the eye (t = −2.739, *p* = 0.006) or at the side of the eyes (t = −6.788, *p* < 0.001, effect size = 0.15) were associated with significantly shorter LOS, i.e., by about 2.1–2.7 d and 3.0–3.8 d, respectively, depending on breed.

Holding both ears erect for the photograph resulted in longer LOS for Labrador retrievers (t = −2.509, *p* = 0.012, effect size = 0.06), but was not statistically significant for Jack Russell terriers or Staffordshire bull terriers. The disadvantage of erect ears was significantly longer for female Labrador retrievers, with a predicted average of 6.7 additional d versus only 0.94 additional d for male Labrador retrievers.

#### 3.2.3. Other Features

The most common photographic background for all breeds was nature (grass/plants/dirt), accounting for 39.6% of Jack Russell terrier photographs, 40.2% of Labrador retriever photographs and 41.1% of Staffordshire bull terrier photographs. Compared with natural backgrounds, indoor (t = −3.528, *p* < 0.001, effect size = 0.08) and kennel structure backgrounds (t = −2.861, *p* = 0.004, effect size = 0.06) were associated with significantly shorter LOS. Indoor backgrounds were especially advantageous for male Staffordshire bull terriers (interaction t = −2.564, *p* = 0.010, effect size = 0.06), who saw an estimated average of 9.8 d shorter LOS when photographed indoors rather than in nature.

The presence of one or more people in the background was associated with shorter LOS (t = −2.578, *p* = 0.010, effect size = 0.06), but the presence of one or more dogs was not (t = 1.122, *p* = 0.262). Corrected for sex and breed, the presence of an accessory such as a collar, bandana, chain, lead or toy in the photograph was associated with significantly longer LOS (t = 9.635, *p* < 0.001, effect size = 0.21), corresponding to an additional average 3.9–4.5 d, depending on breed. There were no significant interactions between accessories and sex or breed. The presence of a visible copyright, suggesting the use of a professional quality photograph, was associated (t = 3.179, *p* = 0.001, effect size = 0.07) with a longer LOS.

## 4. Discussion

This study explored relationships across canine attributes and potential preferences among people seeking to adopt dogs. While the calculated effect sizes are largely less than 0.2 and might be considered small relative to the interquartile range in LOS of approximately 4 weeks, the importance of an effect is determined by a cost-benefit analysis, and in an overcrowded shelter, a few days saved by a more effective marketing of dogs might well result fewer dogs being euthanised per year due to a lack of space, and thus, is not trivial.

Among the four breeds of focal interest in the current study, the photographic representation of Australian cattle dogs appears to have been exceptional. The inordinate number of deletions (n = 1349) among Australian cattle dog profiles is an interesting result in itself. It is unclear why so many photographs of cattle dogs were excluded by our criteria. Study into the unique characteristics of photographs of cattle dogs and the effect that these characteristics have on LOS would be warranted in the future. This finding suggests that, across all Australian rescue and welfare organisations, photographs for this particular breed require improvement. Most of the 1349 deletions were due to blurry photographs or dogs being photographed with their eyes closed. So, ensuring that photographs are clear and appealing is strongly suggested. Indeed, this has been recommended by a previous study of shelter dogs [20]. 

The current LOS results for floppy left ears (15.88 d) and floppy right ears (15.86 d) indicate that dogs with floppy ears have faster adoption rates than dogs with both ears erect (18.32 d). These results were similar to those of Fratkin and Baker [7], who showed that bilaterally floppy-eared dogs were more desirable to potential adopters than pointy-eared dogs. Floppy ears may be a physical aspect of what is known as neoteny, that reflects the typical morphology of young animals and the putative appeal to many humans of puppy-like features [21]. Although Jack Russell terriers with bicoloured coats were the most common of that breed in the current data set, yellow/golden Jack Russell terriers (and their crosses) had the shortest LOS, when corrected for sex. It may be that potential adopters perceived pale coloured dogs as being more friendly or less threatening, as reported by Blecker and colleagues [8]. Their study investigated the effects of human perception and behavioural responses when observing a small or large and pale-coloured or dark-coloured dogs with their owners, and reported that participants regarded large and black dogs as less friendly or potentially dangerous. In the current study, Labrador retrievers with black coat colour were the most common of that breed in the data-set. Black coat colour in dogs has been perceived negatively for decades, not least because of the prevalence of pejorative terms such as “black dog syndrome” within the media [7]. The current study revealed that across all three focal breeds, coat colour did not affect the LOS of dogs, thereby aligning with the results of similar studies [12,22]. It is acknowledged that the interactions of human observer bias with canine coat colour are complex, and may vary with dog size [23].

In contrast to coat colour, the presence of one eye patch had significant effects on LOS for female Labrador retrievers (and their crosses), reducing it by approximately 6.7 d. It would be interesting to explore whether humans have any preference for a patch on the left or the right side of a dog’s face. The coat pattern of white undersides significantly reduced LOS for Jack Russell terriers, i.e., by 4.2 d. Of the 16 photographic variables explored in the current study, nine have not been reported in the literature. When these variables were analysed for associations with LOS and breed, it was found that the effect of eye colour on LOS varied by breed. Dogs with blue/grey iris colour were generally associated with shorter LOS. It may be that people looking for a certain type of may be especially beguiled when they encounter an image of a dog with a blue/grey iris colour. Dogs photographed with mouths open, when corrected for breed and gender, were significantly associated with longer LOS. In particular, Staffordshire bull terriers with this attribute underwent longer LOS than the other breeds in the study, i.e., by 7.8 d. The visibility of lower teeth (hence mouth open) was associated with an additional 5.3 d to LOS. It is possible that to those considering the adoption of a large terrier bred for hunting, the revelation of teeth in a photographic depiction of an unfamiliar dog is off-putting.

Visible sclera was another photographic attribute associated with LOS when corrected for breed and gender. Dogs photographed with the sclera visible at the side, and dogs photographed with the sclera visible at the bottom had the shortest LOS. It is difficult to draw firm conclusions from these results, particularly when various combinations may present completely different pictures. Arguably, dogs with their sclera showing and mouths closed may appear anxious or sad, whereas those with sclera showing and bottom teeth and/or tongue also showing may appear mischievous. Conversely, depending on the observer, certain facial features could denote anxiousness/fear (e.g., tongue curled and sclera visibility) or joy (e.g., mouth open and tongue showing may resemble a “smile” in human expressions). Potential adopters who, simply by choosing to peruse profiles of dogs in need of a home, may have different intentions when acquiring a dog from those who seek puppies who have not yet been in the care of anyone but their breeders.

Of the 16 photographic variables analysed with breed, the photograph’s background was the only variable to have had a significant interaction with both breed and LOS. Jack Russell terriers, Labrador retrievers and Staffordshire bull terriers all had the shortest LOS when photographed in a kennel environment (cage/structure) and the longest when photographed in a natural environment (grass/plants/earth). This seems counterintuitive, and contrasts with previous findings by Thorn and Mitchell [15], who reported that LOS was shorter for dogs photographed outside of kennel environments. In a similar vein, when compared with dogs photographed with their mouths open and tongues showing (e.g., those who might, perhaps anthropomorphically, be described as looking “happier” than dogs who do not), dogs photographed without such attributes had a shorter LOS. It appears that in the current study, dogs shown in austere, rather than natural contexts, may evoke a caring response that might attract prompt adopters. Future studies, perhaps with digital manipulation of the background, may help to establish whether a relatively gloomy portrayal of dogs can increase interest from prospective adopters and how this relates to sympathy. When only people were present in photographs with the dog, dogs had a shorter LOS than otherwise (14.2 d). Dogs in photographs that had been digitally enhanced with texts or frames had a shorter LOS, whereas dogs in professional photographs with a copyright mark visible were associated with a longer LOS. This finding runs counter to our expectations. We had anticipated that dogs would seem more attractive to potential adopters if their profiles were underpinned by professional quality photographs. However, it is possible that, in the case of PetRescue visitors, professional photographs may even suggest the dog is at an advantage over those without professional photographs. The current finding, i.e., that the average LOS is longer for dogs with professional photographs than for those without, may again support the prospect that the potential adopters visiting the site favour dogs that appear to be in need of help. This prospect may be supported when one acknowledges that over 40,000 dogs are euthanised in Australian municipal pounds, shelters and rescue groups annually [24], a figure that may boost pity among some PetRescue visitors and heighten their motivation to save dogs from death. Furthermore, it is recognised that the companion animal adoption process differs between public shelters, nonprofit all-breed shelters, nonprofit all-breed rescue and nonprofit breed-specific rescue organisations [25]. The current findings suggest the future need to distinguish between the motivations of PetRescue visitors and other groups of people acquiring dogs.

The presence of an accessory in the photograph was associated with significantly longer LOS. Collars were associated with the longest LOS. This result contrasts with those of Thorn and Mitchell [15], who explored a sample of 100 dogs and reported that dogs photographed with collars had a shorter LOS than those who did not. Again, this finding points to the possibility that potential adopters visiting PetRescue are looking for a dog to save rather than one who has the accoutrements of an owned dog. However, we cannot leap to the recommendation that dogs should be routinely photographed without collars. Indeed, we note that in most shelter environments, all dogs are required to wear a collar at all times (when outside of kennel) for safety reasons.

It is important to consider the role of relative judgments versus absolute judgments when humans make choices. Humans make different choices when they are evaluating options in isolation compared to when making a relative judgment. If choosing a pet to take home is a relative judgment, this could account for potentially inconsistent findings in previous research. Further research in this domain should consider the roles of attractiveness ratings in humans that have been linked to evolutionary theory [26]. It may also pay to study the eye gaze of observers as they examine images before rating their interest in the adoption of the depicted animal.

## 5. Conclusions

The photographic attributes associated with the shortest LOS in the current study were mouths closed, a black coat colour, floppy ears and being photographed in a kennel structure. The background of photographs was the only variable to have had a significant interaction with breed, in that Jack Russell terriers, Labrador retrievers and Staffordshire bull terriers had the shortest LOS when photographed in a kennel environment. By investigating a range of photographic attributes, we found significant associations between several variables and LOS to reveal the characteristics that prospective owners find appealing when looking at dogs online. Several individual results from the current study contrast with previous studies that suggested that dogs have a better chance of being adopted if they look as though they are owned, rather than living in a rescue shelter. Our results imply that many users of pet adoption sites may actively favour dogs that appear to need help. We suggest that our results may reveal more about the users of these sites than general human tendencies that emerge in response to being presented with photographs of dogs per se. It may be that welfare shelters could use these findings to modify their strategies in order to more effectively rehome animals via online profiles.
